# Engineering a 3D In Vitro Model of Human Gingival Tissue Equivalent with Genipin/Cytochalasin D

**DOI:** 10.3390/ijms23137401

**Published:** 2022-07-03

**Authors:** Cecilia Koskinen Holm, Chengjuan Qu

**Affiliations:** 1 Department of Odontology, Umeå University, 90185 Umeå, Sweden; 2 Wallenberg Center for Molecular Medicine, Umeå University, 90187 Umeå, Sweden

**Keywords:** hTERT-immortalized gingival keratinocytes (TIGKs), hTERT-immortalized gingival fibroblasts (hGFBs), three-dimensional (3D), gingival tissue equivalent (GTE), genipin, cytochalasin D

## Abstract

Although three-dimensional (3D) co-culture of gingival keratinocytes and fibroblasts-populated collagen gel can mimic 3D structure of in vivo tissue, the uncontrolled contraction of collagen gel restricts its application in clinical and experimental practices. We here established a stable 3D gingival tissue equivalent (GTE) using hTERT-immortalized gingival fibroblasts (hGFBs)-populated collagen gel directly crosslinked with genipin/cytochalasin D and seeding hTERT-immortalized gingival keratinocytes (TIGKs) on the upper surface for a 2-week air–liquid interface co-culture. MTT assay was used to measure the cell viability of GTEs. GTE size was monitored following culture period, and the contraction was analyzed. Immunohistochemical assay was used to analyze GTE structure. qRT-PCR was conducted to examine the mRNA expression of keratinocyte-specific genes. Fifty µM genipin (G50) or combination (G + C) of G50 and 100 nM cytochalasin D significantly inhibited GTE contraction. Additionally, a higher cell viability appeared in GTEs crosslinked with G50 or G + C. GTEs crosslinked with genipin/cytochalasin D showed a distinct multilayered stratified epithelium that expressed keratinocyte-specific genes similar to native gingiva. Collagen directly crosslinked with G50 or G + C significantly reduced GTE contraction without damaging the epithelium. In summary, the TIGKs and hGFBs can successfully form organotypic multilayered cultures, which can be a valuable tool in the research regarding periodontal disease as well as oral mucosa disease. We conclude that genipin is a promising crosslinker with the ability to reduce collagen contraction while maintaining normal cell function in collagen-based oral tissue engineering.

## 1. Introduction

Today, there are several three-dimensional (3D) tissue models available, which can be used to study a variety of skin disorders [1,2,3,4]. They are potential alternatives for biological evaluation of both locally and systemically administered drugs, and they provide more relevant information compared to two-dimensional (2D) monolayer culture. Three-dimensional culture systems provide alternative methods to investigate organ behavior by using organoids, and eventually bridge the gap between 2D monolayer culture and animal experiment. Three-dimensional organotypic tissue models offer many advantages over cells in 2D culture since 3D tissue models replicate the differentiated structure and function of native tissue [5,6]. Moreover, the cell environment in 3D culture can be manipulated to stimulate in vivo situation, and provide more precise data in cell-to-cell interaction, tumor characteristics, drug discovery, stem cell research, and disease models [5,6,7].

Biomaterials as an integral component of tissue engineering, have been shown to effectively support cell culture by increasing cell viability and activity [8]. They provide architectural framework of native extracellular matrix for cell growth and tissue regeneration. There are several different biomaterials used to create the scaffolds for the 3D-models. Some of the most frequently used natural polymeric biomaterials such as collagen, alginate, gelatin, chitosan, or hyaluronan have been used in the biomaterial-based regenerative medicine for different tissue engineering due to their high biocompatibility [9,10,11,12]. They exhibit different features that make them suitable in the 3D-modelling of a variety of cells and tissues. Hyaluronan is a glycosaminoglycan that has commonly been used to study biological manners such as cell attachment, growth, and proliferation in different types of tissue repair and regeneration [10]. Gelatin, which is a denatured form of collagen has a high water-solubility rendering in a high permeability of oxygen and nutrients to adjacent cells. It is often used in cancer invasion models [8]. Alginate, on the other hand, is a polysaccharide derived from seaweed. It has mainly been used for cell transportation to damaged tissue due to its ability to incorporate cells. Moreover, it is used for in vitro cell research foremost in stem cells, pancreatic-associated cells or cancer cells [8,9]. Chitosan is a biopolymer that is used as an in vitro scaffold, targeting tissues such as blood vessels, cartilage, bone, and skin [8,11].

Collagen, one of the most important and abundant extracellular matrix protein in mammalian body, has been widely used in tissue engineering, drug delivery, or wound healing. Collagen-based hydrogels have been a preferred choice for skin, bone, cartilage, blood vessels, muscles, and oral mucosa tissue engineering by offering superior biological properties [8]. However, disadvantage of contraction exists in the collagen models [13,14,15,16]. Moreover, the degradation of collagen scaffolds used in tissue engineering is not easily controlled [17,18]. Therefore, covalent crosslinks within and between the constituent collagen molecules have been used to stabilize the collagen construction.

One potential approach in generating tissue constructs for in vitro studies and even for tissue regeneration is to use biomaterials, which are natural to the tissue of interest. Type I collagen is a major component in the dermis of skin and periodontal tissues. Therefore, in efforts to fabricate human constructs, which would closely resemble human equivalent tissue, collagen together with human fibroblasts can be considered to be of utmost importance. Unfortunately, collagen constructs in combination with cells tend to shrink markedly, which results in uncontrolled generation of tissue construct for further use. One essential step obtaining well-regulated formation of skin equivalents is to prevent this shrinking. Generation of crosslinks between the biomaterial nanofibers is one approach to achieve tissue construct, which is more resistant to shrinking.

Genipin is a chemical crosslinking agent for polymers containing amino groups which are abundant in proteins, such as collagen and gelatin. It has been demonstrated to stabilize collagen structure and collagen-based tissue equivalents [19,20,21]. Therefore, it is a good candidate for increasing the stiffness and maintenance of construct shape in hydrogels made of these biomaterials. The crosslinking is able to provide resistance against collagen gel contraction. Indeed, genipin-crosslinked hydrogels have been widely used in the regeneration of skeletal system and different drugs delivery due to its excellent biocompatibility, biodegradability, and stability [22,23,24]. The genipin-crosslinked collagen/gelatin scaffolds or decellularized brain extracellular matrix-gelatin mats have been shown to increase cell proliferation and/or differentiation with different types of cells [20,25,26,27,28,29]. Genipin also has 5000–10,000 times lower cytotoxicity compared to another crosslinker glutaraldehyde that is commonly used for biological tissue fixation [23,30,31]. Importantly, the contraction of 3D skin equivalents can be significantly reduced by genipin crosslinking with a suitable concentration [1]. The hydrogel strength can be readily adjusted by altering genipin concentration, and different types of cells respond differently to genipin [29,32]. All in all, the properties make genipin a good candidate to improve the quality of the 3D constructs.

Cytochalasin D, a cytoskeletal inhibitor, has been demonstrated to inhibit rapid actin polymerization and to induce rapid depolymerization of actin filaments in stimulated platelets [33]. This will prevent the mechanotransduction between microfilaments and integrins, thus inhibiting the cell-based contraction of collagen gel.

Periodontitis, an inflammatory disorder affecting the tooth supporting tissues (the periodontium) has been studied in pre-clinical settings using both animal models and 2D monolayer cultures for many years. The experimental animal models for periodontal diseases have been established in different types of species [34,35,36]. However, the physiopathology in animal models is not fully the same as in humans, which makes it difficult to rely on using only animal models to analyze cellular and molecular mechanisms in human periodontal diseases. Two-dimensional co-culture of bacteria associated with periodontitis and human gingival cells have been used to investigate the mechanisms of periodontal disease [37,38,39]. This model also lacks certain specific cell signals and responses to those signals. Moreover, the 2D monolayer culture system does not adequately mimic the morphological and functional features of the primary tissues. Hence, 3D human gingival tissue equivalents (GTEs) containing gingival fibroblasts and keratinocytes have been established to use in the in vitro research of periodontal disease. Previous studies have shown that 3D GTEs produce more keratinocyte-specific markers, both qualitatively and quantitatively similar to human native gingival tissues rather than 2D cultures [14,40,41].

To the best of our knowledge, we are the first to study the 3D GTE models using genipin-based crosslinked collagen gels. The overall aim of this study is to develop and refine a 3D model suitable for studying cellular events in the soft connective tissue of the periodontium and oral mucosa. The specific aims are to: (1) Construct and validate a practical and physiologically relevant 3D human GTE model using hTERT-immortalized human gingival keratinocytes (TIGKs) and hTERT-immortalized human gingival fibroblasts (hGFBs)-populated collagen gel; (2) further evaluate whether 3D GTEs-containing collagen directly treated with genipin or/and cytochalasin D can be optimized to be more similar to human native gingival tissues compared to uncrosslinked 3D GTEs.

In order to fulfill the aims, we used two types of human cells, including the TIGKs and the hGFBs. They have been co-cultured on either directly crosslinked collagen gel with genipin or/and cytochalasin D for 2-week air–liquid interface culture. Subsequently, the 3D GTEs have been evaluated for their size with macroscopic appearance, cell viability with MTT assay, tissue structure with immunohistochemical (IHC) assay, and mRNA expression of keratinocyte-specific genes with quantitative real time RT-PCR.

## 2. Results

### 2.1. Determination of Noncytotoxic Concentrations of Genipin or Cytochalasin D to the TIGKs and the hGFBs Cultured in 2D Monolayer Cultures

#### 2.1.1. Evaluation of Cellular Metabolic Activity of the TIGKs and the hGFBs

It has been shown that different types of cells can differ in their susceptibility to genipin/cytochalasin D, and skin keratinocytes might be more sensitive to genepin treatment than fibroblasts [4,42]. Therefore, genipin at a concentration ranging from 10–200 µM and 20–400 µM for the TIGKs and the hGFBs, respectively, and cytochalasin D at a concentration ranging from 50–800 nM for both the TIGKs and the hGFBs were selected to evaluate their cytotoxicity in 2D monolayer culture, and to get optimal concentration for the 3D GTE generation.

**Figure 1:**
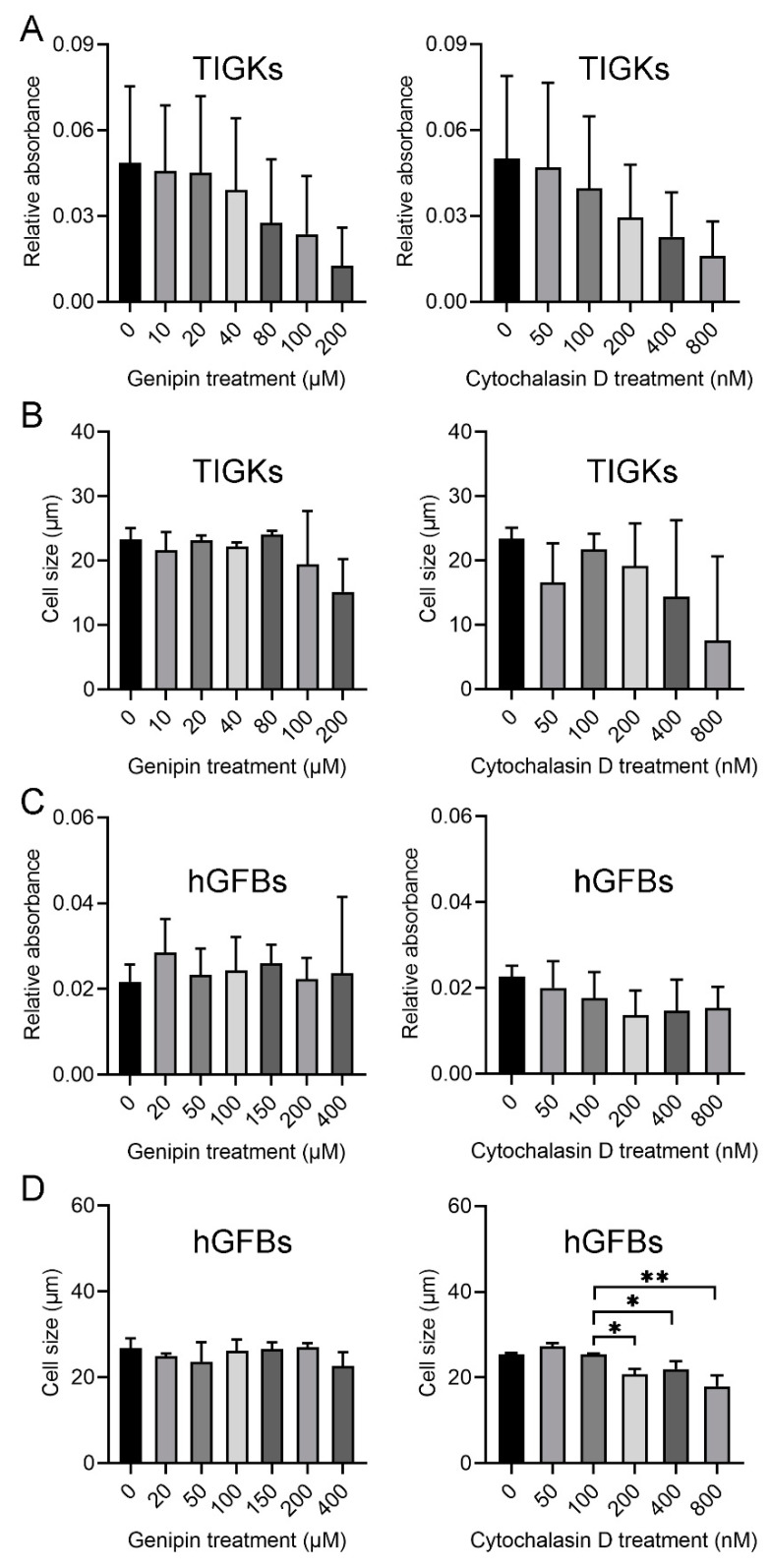
Evaluation of genipin or cytochalasin D toxicity for the TIGKs/hGFBs in the 2D monolayer culture. (**A**). MTT assay of the TIGKs after treatment with different concentrations of genipin or cytochalasin D. (**B**). Cell size of the TIGKs after treatment with different concentrations of genipin or cytochalasin D. (**C**). MTT assay of the hGFBs after treatment with different concentrations of genipin or cytochalasin D. (**D**). Cell size of the hGFBs after treatment with different concentrations of genipin or cytochalasin D. Abbreviations: hGFBs: hTERT-immortalized gingival fibroblasts; TIGKs: hTERT-immortalized gingival keratinocytes. *: *p* < 0.05, **: *p* ≤ 0.001.

MTT assay was used to analyze cell proliferation after the cells were cultured with the selected different concentrations of genipin or cytochalasin D for 48 h. The present results from three separate experiments showed no statistically significant differences in the cell number of the TIGKs between control and different concentrations of genipin (10, 20, 40, 80, 100, and 200 µM) or cytochalasin D (50, 100, 200, 400, and 800 nM), and within the different treatments (Figure 1A). No significant differences were seen among the different treatments (Figure 1A). However, a tendency of decreased number of the TIGKs appeared in the cultures treated with the higher concentration of genipin from 80 µM or cytochalasin D from 200 nM (Figure 1A). Moreover, no statistically significant differences were observed regarding the size of the TIGKs after treatment with either genipin or cytochalasin D (Figure 1B).

Results from the MTT assay did not show any significant differences in the cell number of the hGFBs after treating with different concentrations of genipin (20, 50, 100, 150, 200, and 400 µM) or cytochalasin D (50, 100, 200, 400, and 800 nM) (Figure 1C). However, the size of the hGFBs in the cultures treated with 200, 400, or 800 nM of cytochalasin D appeared statistically significantly smaller than those in control (untreated) and the cultures treated with 50 nM or 100 nM cytochalasin D (Supplementary Table S1, Figure 1D). Therefore, 50 µM genipin (G50) and 100 nM cytochalasin D (C100) were selected in the following 3D constructions.

Moreover, the results from immunocytochemical stainings showed that both TIGKs (Supplementary Figure S1) and hGFBs (Supplementary Figure S2) cultured in the 2D monolayer culture expressed Ki-67 after they were treated with G50 or C100, with no statistical differences compared to control (untreated culture). A very low level of p53 staining appeared in the TIGKs cultured in the 2D monolayer culture, with no significant differences between control and G50- or C100-treated cultures (Supplementary Figure S1). p53 was not detected in the hGFBs after they were cultured in either control conditions or the cultures supplemented with G50 or C100 (Supplementary Figure S2).

#### 2.1.2. Morphological Evaluation of the TIGKs and the hGFBs Cultured in the 2D Monolayer Culture

**Figure 2:**
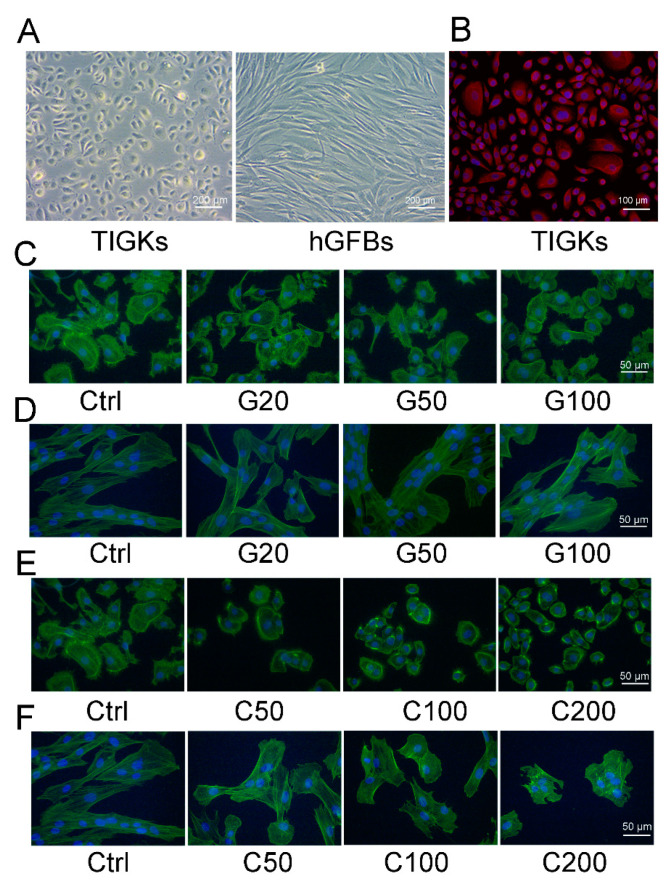
Photomicrographs showing the appearance of the TIGKs/hGFBs in the 2D monolayer culture. (**A**). Phase-contrast microscopic image of the TIGKs and the hGFBs. (**B**). Cytokeratin 14 (K14) staining of the TIGKs (in red). (**C**). Actin staining of the TIGKs (in green) after treatment with genipin. (**D**). Actin staining of the hGFBs (in green) after treatment with genipin. (**E**). Actin staining of the TIGKs (in green) after treatment with cytochalasin D. (**F**). Actin staining of the hGFBs (in green) after treatment with cytochalasin D. Nuclear DNA was labelled with DAPI (shown in blue) in (**B**–**F**). Abbreviations: hGFBs: hTERT-immortalized gingival fibroblasts; hTIGKs: hTERT-immortalized gingival keratinocytes. Ctrl: control; G20: 20 µM genipin; G50: 50 µM genipin; G100: 100 µM genipin, C50: 50 nM cytochalasin D; C100: 100 nM cytochalasin D; C200: 200 nM cytochalasin D.

The TIGKs showed a rounded cobblestone appearance, and the hGFBs had a spindle-shaped morphology when they were cultivated in the 2D monolayer culture (Figure 2A). Cytokeratin 14 (K14) was highly expressed in the TIGKs (Figure 2B) when cultured in the 2D monolayer culture, but neither K10 or involucrin was detected (data not shown). Phalloidin staining showed that genipin- or cytochalasin D-treated TGIKs resulted in disintegration of stress fibers, and ester-like aggregates of short filaments following the increased concentration of genipin or cytochalasin D (Figure 2C,E). Similar appearance was also observed in genipin- and cytochalasin D-treated hGFBs (Figure 2D,F), especially in C100- and C200-treated cultures (Figure 2F). Moreover, the hGFBs completely lost their spindle-shape, and had an oval-shaped morphology in cytochalasin D-treated cultures compared to the spindle-shape in the control (Figure 2F). Genipin or cytochalasin D caused dramatic change in the shape of the TIGKs/hGFBs following the increased concentration of the treatment, and consequently the cells were markedly contracted.

### 2.2. Effects of Genipin or/and Cytochalasin D on 3D Gingival Tissue Equivalents

It is well-known that collagen gels contract after addition of the cells, which causes a significant decrease in the size of cells-populated collagen gel [30,31,43]. Therefore, a suitable crosslinking to collagen gel will not only retain their original shape, but also stabilize the gel scaffold with a favorable cell survival and function.

#### 2.2.1. Positive Effects of Genipin or Combination of Genipin and Cytochalasin D on the Contraction of the 3D Gingival Tissue Equivalent

**Figure 3:**
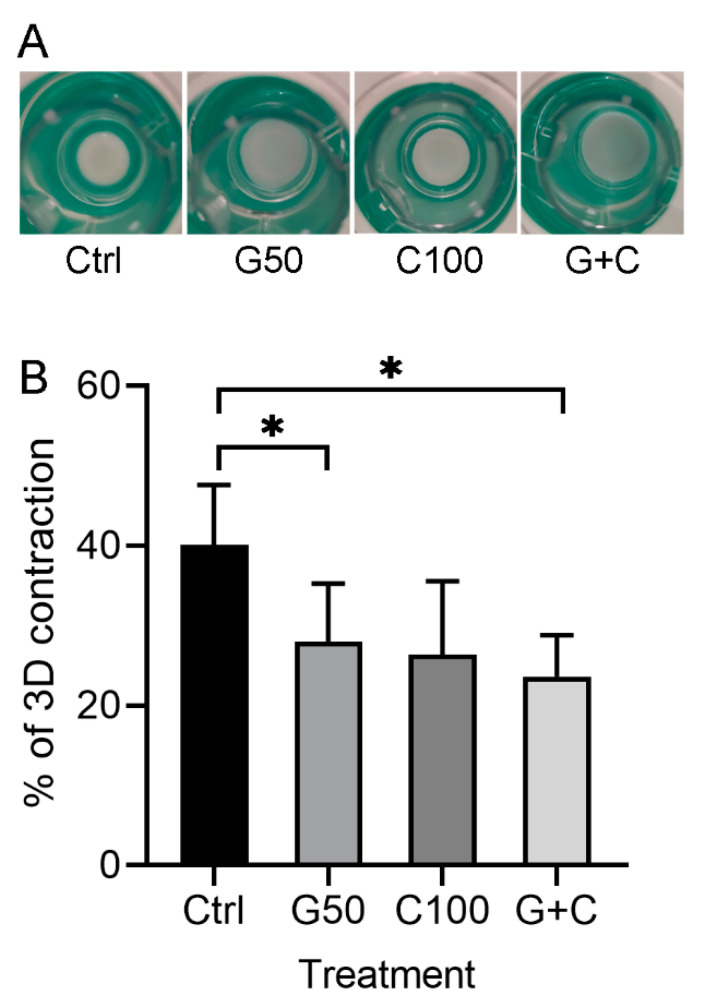
Macroscopic appearance and contraction of the 3D GTEs generated with the TIGKs and the hGFBs-populated collagen gels with/without genipin or/and cytochalasin D direct crosslinking for 2-week air–liquid interface culture. (**A**). Macroscopic appearance of the 3D GTEs by the 2-week air–liquid interface culture. (**B**). Contraction (%) of the 3D GTEs after 2-week airiquid interface culture. Abbreviations: hGFBs: hTERT-immortalized gingival fibroblasts; TIGKs: hTERT-immortalized gingival keratinocytes. Ctrl: control; G50: 50 µM genipin; C100: 100 nM cytochalasin D; G + C: G50 + C100. *: *p* < 0.05.

The 3D GTEs in the present study were constructed according to the workflow as shown in the following method section. Macroscopical appearance showed that the GTEs crosslinked with G50 or in combination of G50 and C100 (G + C) were bigger than that in control (uncrosslinked) (Figure 3A), indicating that the crosslinked collagen gel with G50 alone or G + C significantly inhibited the GTEs contraction compared to the control (Figure 3B). Moreover, the GTEs generated from G50 or G + C crosslinked collagen gel showed opaque and blue color compared to a clear white color in control and C100 crosslinked GTEs (Figure 3A). The GTE treated with C100 was a bit larger than that in control, but smaller than that in G50 or G + C-crosslinked GTEs (Figure 3A). However, they did not reach a statistical significance (Figure 3B).

#### 2.2.2. Effects of Genipin or/and Cytochalasin D on Cellular Metabolic Activity in the 3D Gingival Tissue Equivalents

**Figure 4:**
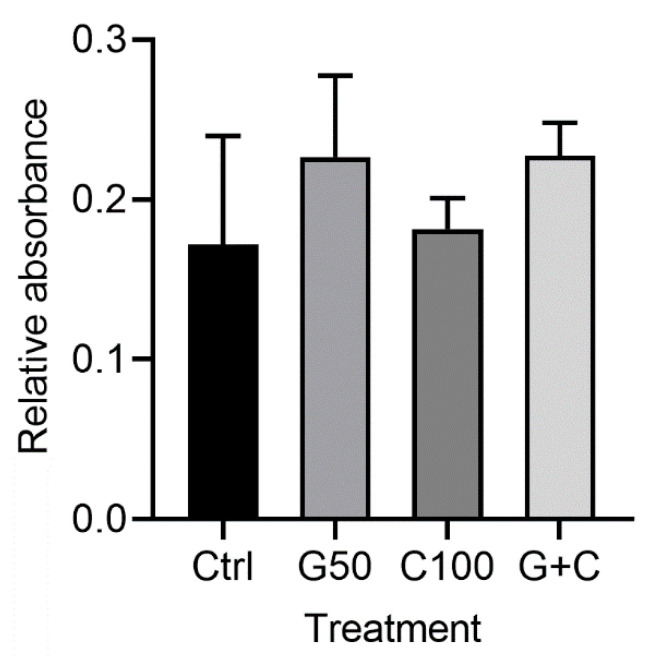
MTT assay of the 3D GTEs generated from the TIGKs and the hGFBs-populated collagen gels with/without genipin or/and cytochalasin D crosslinking in 2-week air–liquid interface culture. Abbreviations: hGFBs: hTERT-immortalized gingival fibroblasts; TIGKs: hTERT-immortalized gingival keratinocytes, GTE: gingival tissue equivalent; Ctrl: control; G50: 50 µM genipin; C100: 100 nM cytochalasin D; G + C: G50 + C100.

MTT assay of the 3D GTEs did not show any statistically significant differences regarding the cell number between the control and the treated cultures or among the treated cultures (Figure 4). Although the GTEs-crosslinked with G50 or G + C showed a higher cell number compared to the control, it did not reach a statistically significant difference (Figure 4).

#### 2.2.3. mRNA Expression of Keratinocyte and Fibroblast Genes in the 3D Gingival Tissue Equivalents

Quantitative RT-PCR was used to analyze the mRNA expression of keratinocyte-specific genes [cytokeratin 14 (K14), cytokeratin 10 (K10) and involucrin)], and fibroblast-associated genes (collagen 1a1 and CD90) in the 3D GTEs generated with the TIGKs and the hGFBs-populated collagen gels with or without genipin or/and cytochalasin D treatment after 2-week air–liquid interface cultures.

**Figure 5:**
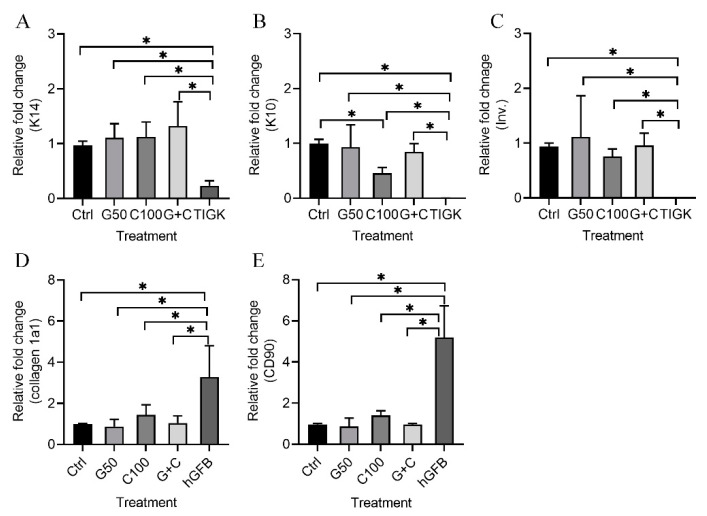
Quantitative RT-PCR assay [(**A**) = K14, (**B**) = K10, (**C**) = Involucrin (Inv.), (**D**) = collagen 1a1 and (**E**) = CD90] of the 3D GTEs generated with the TIGKs and the hGFBs-populated collagen gels with/without genipin/cytochalasin D direct crosslinking for 2-week air–liquid interface culture. Abbreviations: hGFBs: hTERT-immortalized gingival fibroblast; TIGKs: hTERT-immortalized gingival keratinocytes. Ctrl: control; G50: 50 µM genipin; C100: 100 nM cytochalasin D; G + C: G50 + C100. *: *p* < 0.05.

The present results show that mRNA expression levels of K14 (Figure 5A), K10 (Figure 5B), and involucrion (Figure 5C) in all the 3D GTEs (Ctrl, G50, C100, and G + C) were statistically significantly higher compared to those in the TIGKs-cultured in the 2D monolayer culture. K10 mRNA expression levels in the 3D GTEs generated with C100 treated hGFBs-populated collagen gels were significantly lower than those in the 3D GTEs generated from control (Figure 5B). However, no statistically significant differences were detected in the mRNA expression levels of K14 (Figure 5A) and involucrin (Figure 5C) of the 3D GTEs generated from either uncrosslinked or crosslinked hGFBs-populated collagen gels. Moreover, no significant differences were detected in the mRNA expression levels of K10 in the 3D GTEs generated from control and G50 or G + C (Figure 5B).

The mRNA expression levels of collagen 1a1 (Figure 5D) and CD90 (Figure 5E) were significantly lower in the 3D GTEs generated from the crosslinked or uncrosslinked hGFBs-populated collagen gels than those in the hGFBs cultured in the 2D monolayer culture.

#### 2.2.4. Histological Evaluation of the 3D Gingival Tissue Equivalents

**Figure 6:**
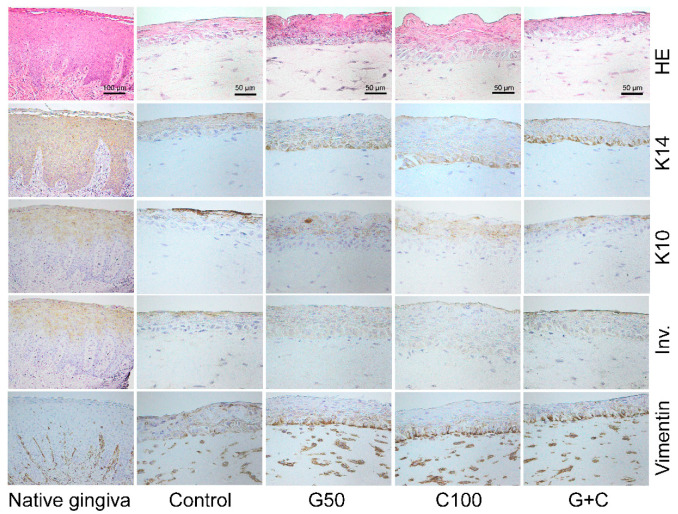
Immunohistochemical staining (5-µm section) of HE, K14, K10, Inv., and vimentin in human 3D gingival tissue equivalents (GTEs) generated with TIGKs and hGFBs-populated collagen gel with/without genipin/cytochalasin D direct crosslinking for 2-week air–liquid interface culture. Abbreviations: hGFBs: hTERT-immortalized gingival fibroblasts; TIGKs: hTERT-immortalized gingival keratinocytes; G50: 50 µM genipin; C100: 100 nM cytochalasin D; G + C: G50 + C100; HE: hematoxylin & eosin; K14: keratin 14; K10: keratin 10; Inv.: involucrin.

The histological analyses were used to study the tissue structure of the 3D GTEs. Hematoxylin and eosin (HE) stainings showed that the morphology of the TIGKs and the hGFBs in our 3D GTEs generated with the TIGKs and either crosslinked or uncrosslinked hGFBs-populated collagen gels were similar to those in the human native gingival tissue (Figure 6). The histological evaluation demonstrated the collagen hydrogel populated with hGFBs, and the adherence of the seeded TIGKs on top of the collagen gels, as well as development of a multilayered stratified epithelium on its surface (Figure 6). Moreover, the histological sections from the 3D GTEs generated with the TIGKs and either genipin or cytochalasin D treated hGFBs-populated collagen gels exhibited clear suprabasal and basal layers in the epithelium, similar to human native gingiva, compared to control (Figure 6).

Immunohistochemical staining showed a stronger expression of K14 in basal layer, and lower expression in suprabasal layer of the 3D GTEs generated with the TIGKs and genipin/cytochalasin D treated hGFBs-populated collagen gels compared to control (Figure 6). Expression of K10 and involucrin were detected in fully differentiated epithelium of the 3D GTEs generated with the TIGKs and either uncrosslinked or crosslinked hGFBs-populated collagen gels, which is similar to human native gingival tissue (Figure 6). Vimentin-positive hGFBs were detected in the connective tissue of all 3D GTEs generated with the TIGKs and either uncrosslinked or crosslinked hGFBs-populated collagen gels, as well as in human native gingival tissue (Figure 6). There were no clear differences visible in the vimentin stainings in the connective tissue of the 3D GTEs generated from either uncrosslinked or crosslinked collagen gels, or among the crosslinked collagen gels (Figure 6). Moreover, the expression of Ki-67 in the basal layer was clearly detected in all 3D GTEs generated from either genipin/cytochalasin D alone or combination of G50 and C100 crosslinked collagen gels, compared to the control (Supplementary Figure S3). On the other hand, p53 was not detected in the 3D GTEs generated from uncrosslinked or crosslinked collagen gels (Supplementary Figure S3).

## 3. Discussion

Tissue constructs consisting of gingival fibroblasts in collagen gel with differentiated keratinocytes on top of the collagen gel would offer an excellent 3D model for in vitro studies of gingival tissue, and would lay basis for the fabrication of tissue grafts applicable even for tissue regeneration. However, there are technical problems today, which limit the usability of the in vitro fabricated grafts. In particular, the uncontrolled contraction of the collagen gels generally restricts their value for the above-mentioned purposes. The development of protocols, which better allow the fabrication of constructs with desired size and native kind of cellular content and architecture is highly valuable.

In this study, we describe a culture protocol for full-thickness human GTE generated with the hGFBs-populated collagen gel, directly treated with genipin/cytochalasin D, and the TIGKs. We demonstrate that our human GTE expresses keratinocyte-specific genes, and shows phenotypical similarities to the human native gingival tissue. Genipin alone or in combination with cytochalasin D significantly reduced the contraction of hGFBs-populated collagen gel without damaging the epithelium layer. Thus, the developed culture protocol improves our possibility to obtain multilayered tissue constructs with differentiated keratinocytes on top of fibroblast embedded in collagen gels.

Primary gingival cells would be ideal cells for generation of the GTE constructs. However, they have a finite lifespan that limits their proliferative capacity to provide sufficient cell numbers for use in 3D organotypic cultures. Therefore, the unlimited supply of the immortalized cells with normal primary cell-like properties were used in our 3D cultures. In the present study we found that the TIGKs and the hGFBs used in the GTEs generation showed same morphological appearance as primary keratinocytes and fibroblasts cultured in 2D monolayer culture. The TIGKs strongly expressed keratinocyte-specific marker, K14. Moreover, the 3D GTE generated with the TIGKs and the hGFBs expressed significantly higher levels of K14, K10, and involucrin compared to the TIGKs cultured in the 2D monolayer culture. Therefore, we can assume that the hTERT-immortalized keratinocytes are a good alternative cell source in the 3D gingival models. The 3D GTE generated with immortalized cells mimics the natural microarchitecture of extracellular matrix, which improves the cell proliferation and differentiation. Similarly, some previous studies have also successfully developed full-thickness 3D GTE models using immortalized primary keratinocytes and fibroblasts [32,40,44]. It has also been done for 3D human skin equivalent [2,3].

However, the contraction of the collagen gel in the 3D GTEs in above mentioned studies, has not been well-described, even though the contraction indeed exists. It has been shown that the continuous contraction of the skin equivalent during the experimental procedure results in unstable collagen gels as well as degradation of the collagen. This further impairs the epidermal layer formation, and the epidermal and dermal junction [1], as well as cell behavior [45,46]. The initial collagen concentration and final number of fibroblasts in the collagen gel have significant effects on the contraction of collagen gel, and further alter the engineered tissue structure [47,48]. Therefore, we performed a pilot study to optimize a suitable hGFB number for the 3D GTE generation to avoid unnecessary contraction. Subsequently, the optimal cell density of 0.325 × 10^6^ hGFBs/mL was used in the 3D GTE generation.

In tissues, collagen fibers have intra- and interfibrillar crosslinks, which affect their mechanical properties and control the deformation of the tissue. Thus, the content of crosslinks can be modified to restrict the contraction of collagen gels. Genipin is an excellent natural crosslinker, which can spontaneously react with the amines in amino acids and proteins to form water-soluble blue pigments [49]. The reaction between genipin and collagen induces the formation of cyclic structures, which enable intramolecular and intermolecular crosslinks [19]. Genipin has successfully been used to crosslink collagen in 3D skin equivalent, in which genipin effectively reduces the collagen contraction. Furthermore, genipin solution at the concentration of 0.4 mmol/L has a very low cytotoxicity for the cells in the fibroblasts-populated collagen gel [1]. It has also been shown that genipin at concentrations of 10 µM and below has no cytotoxic effect on skin keratinocytes [4]. However, genipin at the concentrations above 150 µM shows cytotoxic effect to skin fibroblasts [4]. The advantage of genipin is that the strength of hydrogel can be readily adjusted by altering the genipin concentration [4,12,23].

In the present study we mixed the genipin solution into the hGFBs-populated collagen gel to see whether the direct crosslinker genipin to the mixture of hGFBs-collagen could sufficiently reduce the collagen contraction while maintaining good cell behavior. The optimal genipin concentration used in the present GTEs was optimized from 2D and 3D cultures. We are able to show that the TIGKs and the hGFBs had comparable cell proliferative capacity in 50 µM genipin treatment compared to controls. Additionally, 50 µM genipin treatment resulted in cell morphological changes compared to the control, but the cell size was unaffected. The contracted TIGKs and the hGFBs exhibited 3D-shape compared to the control. The findings might be explained, due to the decrease in cell adhesion area and increase in cell height after the genipin treatment. This could further promote keratinocyte differentiation and formation of epithelium. It has been shown that genipin-crosslinked type II collagen scaffold caused morphological change of adipose-derived stem cells (ADSCs) from spread shape toward spherical shape, and promoted proliferation and differentiation of ADSCs into nucleus pulposus-like cells via the activation of Shh signaling pathway [28].

It has been shown that genipin crosslinking at 0.4 mmol/L effectively reduced the contraction of 3D skin equivalent, and increased its stability [1]. Similar to our present results that the contraction of the 3D GTEs was significantly reduced after treating with G50 or G + C crosslinking compared to control (uncrosslinked), they also showed comparable cell proliferation capacity with respect to uncrosslinked GTEs. A well-developed multilayered stratified epithelium with a clearly visible suprabasal and basal layer from our histological samples further confirmed that genipin crosslinking did not impair the epithelium layer formation, but improved the epithelium-connective tissue junction in the GTE.

We also found that our 3D GTEs generated with G50 or G + C crosslinking appeared in blue color when compared to clear color of the GTEs generated from control or C100 treatment. This result confirmed that in our model, genipin reacted with amines in amino acids and proteins and produced crosslinks, which formed water-soluble blue pigments. It has been shown that the genipin-mediated crosslinking produced significant concentration- and incubation time-dependent increases fluorescence intensity, which strongly correlated with the stiffness of collagen [50]. Previous studies have shown that the genipin-crosslinked collagen increased mechanical strength in porcine sclera with a concentration- and incubation-dependent manner [51]. Accordingly, we assume that the stiffness of our 3D GTEs generated from G50 and G + C crosslinking might be stronger than those GTEs generated from uncrosslinking and C100 treatment.

In the present study, we also used cytochalasin D-treated collagen to examine whether this cytoskeletal disruptor had any advantages in the generation of the 3D GTEs while inhibiting the integrins-microfilament-related mechanotransduction, which potentially leads to the collagen contraction. Although cytochalasin D has been widely used to disrupt actin filaments in many cell types, its sufficient and non-cytotoxic concentrations vary in different cell types. Cytochalasin D at a concentration ranging from 3.1–800 nM inhibited the contraction of dog periodontal ligament fibroblasts-incorporated collagen gel in a dose-dependent manner, and 800 nM of cytochalasin D could completely inhibit the contraction [42]. Cytochalasin D at 20 µM completely disrupted actin filament in rabbit patellar fibroblasts [52]. We found that cytochalasin D with the concentration higher than 100 nM had obvious negative effects on the proliferation of the TIGKs and the hGFBs in the 2D monolayer cultures, especially for the TIGKs. We also found that the shape of the TIGKs and the hGFBs changed toward nearly round-shape after they were cultured with 100 nM cytochalasin D. This is due to the disruption of stress fibers by cytochalasin D, and results in decreased cell adhesion area and increased cell height. It has been shown that cytochalasin D stimulated chondrogenesis of fetal rat chondrocytes with modification of cytoskeleton structure and subsequent rounding changes of the cells [53]. Thus, we selected 100 nM cytochalasin D in the 3D generation. We found that 100 nM of cytochalasin D produced a good multilayered epithelium with clear suprabasal and basal layers. It expressed keratinocyte-specific markers, similar to 50 µM genipin crosslinked GTEs. However, it did not effectively reduce the collagen contraction compared to genipin, neither effectively preserved the cell proliferation. Cytochalasin D at 100 nM concentration has been shown to significantly reduce the contraction of fetal mice mesenchymal stem cells/derma fibroblasts-populated collagen gels in low cell density [54].

We also used the combination (G + C) of G50 and C100 to see if the crosslinking of this combination could produce the GTEs with better multilayered stratified epithelium than genipin or cytochalasin D alone. Although we did not see the anticipated outcome, we saw that the 3D GTEs generated from either genipin/cytochalasin D alone or combination of genipin and cytochalasin D crosslinked collagen gels clearly expressed the cell proliferation marker Ki-67 in the basal layer of epithelium compared to the control. But no expression of the apoptotic marker p53 was detected in the 3D GTEs. Dermal fibroblasts have been reported to positively affect the keratinocytes proliferation, adhesion, and differentiation as well as improved epidermal morphology [55,56,57,58].

Connective tissue fibroblasts exhibit motility and contractility, which play a central role during connective tissue formation and remodeling, and wound repair. They are the principal cell type in the periodontal connective tissues. Their ability to proliferate, migrate, adhere, elongate, immobilize itself and begin matrix synthesis is essential for cell function, wound healing and tissue integrity [59]. It has been shown that keratinocyte-released factors regulate the fibroblasts to act catabolically on the extracellular matrix in the epithelialization processes in organotypic culture model by the down-regulation of collagen synthesis and up-regulation of matrix metalloproteinase [60,61]. Keratinocyte-derived cytokines have been shown to decrease type I collagen mRNA expression in co-culture of the keratinocytes and the fibroblasts compared to monoculture of the fibroblasts [62,63]. Our results with the mRNA expression of collagen 1a1 and CD90 in the 3D GTEs further confirmed that the fibroblasts in the lamina propria of gingival connective tissue secret and organize discrete collagen networks and support the formation of epitheliums. Thereby, the keratinocytes have an antifibrotic effect on the fibroblasts.

A limitation of this study is that the multilayered stratified epithelium of the GTEs produced with the TIGKs and G50 or G + C directly crosslinked hGFBs-populated collagen gel lack of rete pegs, which are projections of the epithelium into connective tissue in natural oral mucosa of the attached gingivae. They provide better mechanical resistance and nutritional supply between epithelium and lamina propria [64]. Absence of rete pegs in the GTEs could cause the epithelium to easily detach, and thereby limits its clinical application. To overcome this issue by properly adding mechanical pressure or ERK1/2 activators, such as EGF or estrogen, during epithelium development in GTE could increase epidermal extension and branching into connective tissue.

## 4. Materials and Methods

### 4.1. Cell Culture

hTERT-immortalized gingival keratinocytes (TIGKs, CRL-3397, ATCC) were grown in CnT-07 (Cellntec) culture medium till 80–90% confluent before the experiments were conducted. hTERT-immortalized gingival fibroblasts (hGFBs, CRL-4061, ATCC) were grown in fibroblast medium that contains Dulbecco’s modified eagle medium (DMEM, Gibco) supplemented with 10% fetal bovine serum (FBS, Gibco) and 1% penicillin/streptomycin (Gibco) till 80–90% confluent before the experiments were started. During the expansion culture of the TIGKs or the hGFBs in the 2D monolayer, the medium changed every other day.

The TIGKs were cultured on coverslips in 24-well plate (142,475, ThermoFisher Scientific, Waltham, MA, USA) to 60% confluence. Then the paraformaldehyde (PFA)-fixed cells were incubated with K14 (1:200, LL002, Thermofisher Scientific) at 4 °C overnight after permeabilization with 0.1% Triton X-100 (108,603, Merck, Readington Township, NJ, USA). Next day the cells were incubated with related secondary antibody (Texas red anti-mouse, TI-2000, Vector laboratories, Newark, CA, USA) for 1 h at room temperature after washing. Subsequently, the cell nuclei were stained with 1 µg/mL 4′,6-diamidino-2-phenylindole (DAPI, D9542, Sigma-Aldrich, St. Louis, MO, USA). Finally, the stained cells were mounted on a glass slide with Antifade mounting medium (H-1800, Vector laboratories), and photographed with fluorescence microscope (Olympus BX41).

### 4.2. Detection of Genipin/Cytochalasin D Toxicity on the TIGKs and the hGFBs in the 2D Monolayer Culture

The cell viability and proliferation were detected by MTT colorimetric assay. Briefly, the TIGKs or the hGFBs with cell density of 50,000 cells/well were treated with different concentrations of genipin (G4796, Sigma-Aldrich) (10, 20, 40, 80, 100, and 200 µM) or cytochalasin D (C2168, Sigma-Aldrich) (50, 100, 200, 400, and 800 nM) in CnT-07 medium or fibroblast medium, and cultured in 12-well plate (150,628, Thermofisher Scientific) for 48 h. By the end of culture, the cells were washed with PBS, subsequently incubated with 2 mL of 0.5 mg/mL MTT reagent 3-(4,5-dimethylthiazol-2-yl)-2,5-diphenyltetrazolium bromide (M2128, Sigma-Aldrich) in 37 °C incubator for 3 h. Then the MTT formazan salt was dissolved in 1 mL of dimethylsulphoxide/ethanol (1:1, *v*/*v*), and the absorbances were measured at 595 nm with a 96-well plate reader (82.1581, Sarstedt, Newton, NC, USA). Three replicates for every sample were used in the measurement, and the measurement was repeated three times from three different cell experiments.

The size of the TIGKs and the hGFBs were measured with Countess automated cell counter (Invitrogen, Waltham, MA, USA) after cultivation at different concentrations of genipin or cytochalasin D for 48 h in the 2D monolayer culture. The measurements were repeated three times from three different cell cultures.

### 4.3. Actin Filament Staining of the TIGKs or the hGFBs in the 2D Monolayer Culture

The TIGKs or the hGFBs were cultured on coverslip in a 24-well plate at different concentrations of genipin (20, 50 or 100 µM) or cytochalasin D (50, 100 or 200 nM) to 60% confluence. Then the PFA-fixed cells were incubated with phalloidin (1:200, Alexa Fluor^TM^488, A12379, Thermofisher Scientific) after permeabilization with 0.1% Triton X-100. Subsequently, the cells were incubated with DAPI (1 µg/mL) for 10 min at 37 °C after washing with PBS. Finally, the phalloindin-stained cells were photographed with fluorescence microscope after mounted on a glass slide with Antifade mounting medium.

### 4.4. Immunocytochemical Staining of the TIGKs/hGFBs with Ki-67 and p53 in the 2D Monolayer Culture

The TIGKs and the hGFBs were cultured on coverslips in a 24-well plate with 50 µM genipin or 100 nM cytochalasin D for 48 h. Then the cells were fixed with 4% PFA for 20 min after washing with PBS. Subsequently, the fixed cells were incubated with either Ki-67 (1:100, Sc-23900, Santa Cruz biotechnology, Dallas, TX, USA) or p53 (1:100, Sc-46978, Santa Cruz Biotechnology) overnight at 4 °C after permeabilization with Triton X-100. The next day after washing, the cells were incubated with secondary antibody (1:200, Horse anti-mouse IgG antibody (H + L), Fluorescein, FI-2000, Vector Laboratories) for 1 h at room temperature. Then the cells were incubated with DAPI for 10 min at 37 °C after washing with PBS. Finally, the cells were photographed with fluorescence microscope after mounted on a glass slide with Antifade mounting medium.

### 4.5. Construction of the 3D Gingival Tissue Equivalents

**Figure 7:**
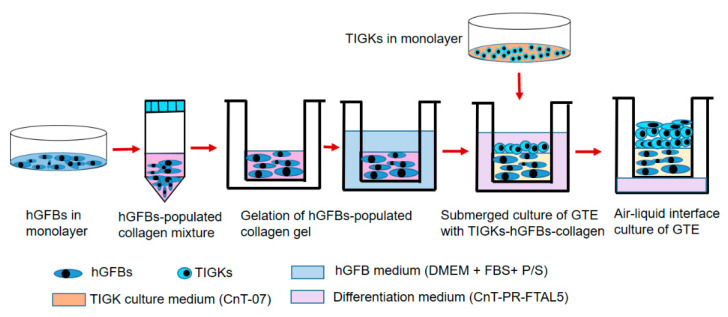
Workflow chart showing the procedure of gingival tissue equivalent (GTE) generation. Abbreviations: hGFBs: hTERT-immortalized gingival fibroblasts; TIGKs: hTERT-immortalized gingival keratinocytes.

The GTEs were constructed by preparation of the collagen mixture with collagen I rat tail (4 mg/mL. Collagen G, L7213, Sigma-Aldrich), adding the hGFBs to the collagen mixture, and loading the TIGKs to top of the hGFBs-populated collagen scaffold step by step (Figure 7). Briefly, the collagen mixture was prepared by mixing eight parts type I collagen solution and one part 10x HBSS, and adjusted pH to 7.5 with dropwise addition of 1 M NaOH. The hGFB-collagen mixture was prepared by adding one part of FBS with a designed amount of the hGFBs (0.325 × 10^6^/mL) to the collagen mixture. Then 50 µM of genipin, 100 nM of cytochalasin D or the combination (G + C) of G50 + C100 crosslinked hGFBs-populated collagen gel was prepared by directly adding genipin or/and cytochalasin D solution to the hGFBs-populated collagen mixture. The hGFBs-populated collagen mixture was kept on ice during the whole procedure. Subsequently, 1 mL of the hGFBs-populated collagen mixture were carefully loaded to a transwell insert of 12-well insert (353,292, Thermofisher Scientific), and gelation was allowed to proceed in 37 °C for two hours. Thereafter, the hGFBs-populated collagen gel was incubated in 37 °C with 5% CO_2_ for one hour after adding 1 mL of fibroblast medium per insert, and 1.5 mL fibroblast medium to the well plate of 12-well plate (353,043, Thermofisher Scientific). Finally, 0.9 × 10^6^ TIGKs were loaded on top of the hGFBs-populated collagen gel, and the GTE construct with the TIGKs-hGFBs-collagen gel was incubated submerged for three days with differentiation medium, CnT-PR-FTAL5 (Cellntec). After three days, the insert with the GTE constructs were cultured in air–liquid interphase culture with CnT-PR-FTAL5 for two weeks (Figure 7). The culture medium was changed every two days.

### 4.6. Cytotoxicity Analysis of the 3D Gingival Tissue Equivalents

The genipin or cytochalasin D cytotoxic effects on the GTEs were analyzed using MTT assay by the end of 3D culture after 2-week air–liquid interface culture. Briefly, the constructs with the TIGKs-hGFBs-collagen gels were immersed in 1 mL MTT (1.15 mg/mL) in PBS at 37 °C for three hours. Then the cells and the GTEs were incubated with 1.5 mL acidified isopropanol (20,842.312, VWR, Radnor, PA, USA) for one hour at room temperature with stirring. The extraction was measured at an absorbance of 570 nm on a spectrophotometer (Multiskan GO, Thermofisher scientific).

### 4.7. Histological and Immunohistological Assay of the 3D Gingival Tissue Equivalents

By the end of culture, the GTEs were fixed in 4% PFA overnight at 4 °C. Next day, the fixed tissues were dehydrated with a series of gradually increasing concentrations of ethanol, and embedded in Tissue-Tek III embedding paraffin. After dewaxing and rehydration, tissue sections (5 µm) were stained with HE for histological examination. The sections were processed for IHC to study the expression of specific proteins. After antigen retrieval by citrate buffer, the sections were blocked with 5% FBS, and then incubated overnight at 4 °C with corresponding primary antibodies against human K14 (1:400), K10 (1:2500, ab76318, Abcam, Cambridge, UK), involucrin (1:250, NB100-2727 SY5, Novus biological, Thermofisher Scientific), Ki-67 (1:50, P6834, Sigma-Aldrich), p53 (1:100, Sc-47698, Santa Cruz Biotechnology), vimentin (1:500, EPR3776, Abcam). On the following day, the sections were incubated with HRP-labelled secondary antibodies (polyclonal Goat anti-Rabbit immunoglobulins/HRP, P0448, Dako; Goat anti-Mouse IgG(H + L), HRP, 31430, Thermofisher Scientific) for one hour at room temperature. After the PBS washes, the sections were incubated with DAB substrate (ab64238, Abcam) for 10 min followed by hematoxylin staining. Microscopic slides were visualized and recorded with Olympus BX41 microscope.

### 4.8. Quantitative Reverse Transcription Polymerase Chain Reaction (qRT-PCR)

Total RNA was extracted from the 3D GTEs using Trizol reagent according to the manufacturer’s instructions after tissue homogenization with bullet blender (Techtum). The RNA was quantified with a Nanodrop-2000c spectrophotometer (ThermoFisher Scientifc), and complementary DNA (cDNA) was synthesized using Ultrascript 2.0 cDNA synthesis kit (PB30.31-10, PCRBIOSYSTEM) with 1 μg of RNA in 20 μL reaction volume. Each 10 μL RT-PCR reaction contained 4 μL (4 μg) of cDNA, 6 μL of 2x qPCRBIO SyGreen Mis Lo-ROX (PB20-11, PCRBIOSYSTEM), and forward and reverse primers for K14, K10, Involucrin, collagen 1a1, CD90 or ribosomal protein Large P0 (RPLP0) (400 nM). The sequences of the primer pairs are shown in Table 1. The qRT-PCR analyses of these genes were performed in a Quantstudio Taqman Real-Time PCR System (Applied biosynthesis).

The qRT-PCR reactions were started by heating up the samples to 95 °C for two minutes followed by 40 times repeated cycle of denaturation (95 °C, 5 s) and annealing (60 °C, 30 s). The amplification efficiencies were calculated from standard curves of the qRT-PCR reactions from each primer pair. The relative gene expression levels of the genes, human K10, K14, involucrin, collagen 1a1, and CD90 were calculated with Pfaffl-method [69] by normalization to the housekeeping gene RPLP0. Efficiencies in the range of 90–110% were accepted, and the specificities of the PCR products were determined with 2.5% (*w*/*v*) agarose gel electrophoresis separation. The experiments were performed at least three times from three different cultures.

### 4.9. Statistical Analysis

Statistical analyses were performed using SPSS software, version 27 (IMB SPSS Statistics 27, Chicago, IL, USA). All data from cell experiments were analyzed by mean ± SD from at least three independent experiments. Data were tested for normality using Shapiro–Wilk test, and thereafter, comparison between multiple groups were performed with One-way ANOVA test with LSD post Hoc. For non-parametric data, Kruskal–Wallis H and Mann Whitney were used. *p*-values less than 0.05 were considered statistically significant.

## 5. Conclusions

The TIGKs and the hGFBs-populated collagen gel directly crosslinked with G50 or G + C can generate 3D GTE with a fine multilayered epithelium, which resemble native gingival tissue. Genipin can significantly reduce collagen contraction without damaging the epithelium layer, while maintaining cell proliferative capacity. These results indicate that the TIGKs and the hGFBs can successfully form organotypic multilayered cultures, which can be a valuable tool in the research of periodontal disease, other oral soft tissue diseases, drug discovery, stem cells application, and as an alternative method to animal model. Genipin is a promising crosslinker due to its capacity to reduce collagen contraction while maintaining normal cell function in collagen-based oral tissue engineering.

## Figures and Tables

**Table 1:** The list of human primers used in qRT-PCR.

Gene	Primer Pairs 5′→3′	Product Size (bp)	Reference
RPLP0	F: AGATGCAGCAGATCCGCAT R: GTGGTGATACCTAAAGCCTG	319	[65]
K14	F: CATGAGTGTGGAAGCCGACAT R: GCCTCTCAGGGCATTCATCTC	154	[66]
K10	F: TTGCTGAACAAAACCGCAAAG R: GCCAGTTGGGACTGTAGTTCT	169	[67]
Inv.	F: GACTGCTGTAAAGGGACTGCC R: CATTCCCAGTTGCTCATCTCTC	250	[67]
Coll 1a1	F: GAACGCGTGTCATCCCTTGT R: GAACGAGGTAGTCTTTCAGCAACA	94	[68]
CD90	F: TCGCTCTCCTGCTAACAGTCT R: CTCGTACTGGATGGGTGAACT	134	[65]

F: forward primer; R: reverse primer; RPLP0: ribosomal protein, large, P0; K14: cytokeratin 14; K10: cytokeratin 10; Inv: involucrin; Coll 1a1: collagen 1a1.

## Data Availability

Not applicable.

## Supplementary Materials

The following supporting information can be downloaded at: https://www.mdpi.com/article/10.3390/ijms23137401/s1.
