# Supplementary material for: Engineering a 3D In Vitro Model of Human Gingival Tissue Equivalent with Genipin/Cytochalasin D
Source: Int J Mol Sci. 2022 Jul 3;23(13):7401. doi: 10.3390/ijms23137401 (PMC9266888; doi:10.3390/ijms23137401)
Supplement: Supplementary file 1 [file ijms-23-07401-s001.zip › ijms-1794101-supplementary.pdf]

Supplementary Figures

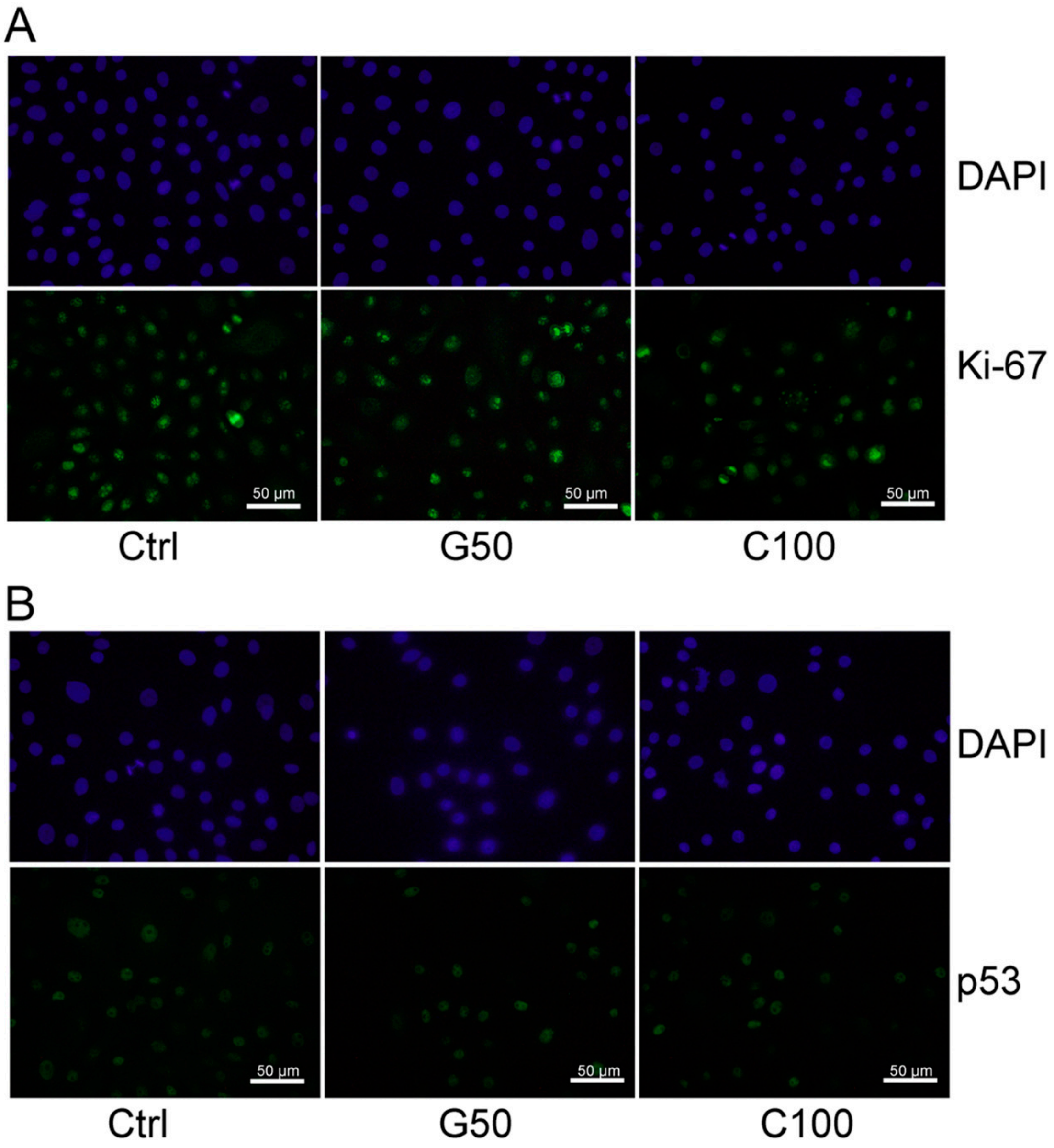

Supplementary Figure S1. Immunostaining of the TIGKs with Ki-67 (**A**) or p53 (**B**). The PFA-fixed TIGKs were immunostained with anti-Ki67 antibody (Ki-67), or anti-p53 antibody (DO-7) after the cells were treated with 50 μM genipin (G50) or 100 nM cytochalasin D (C100) for 48 hours. The cells stained with Ki-67 or p53 show in green colour, while nuclei are stained in blue with DAPI. Ctrl: control (untreated).

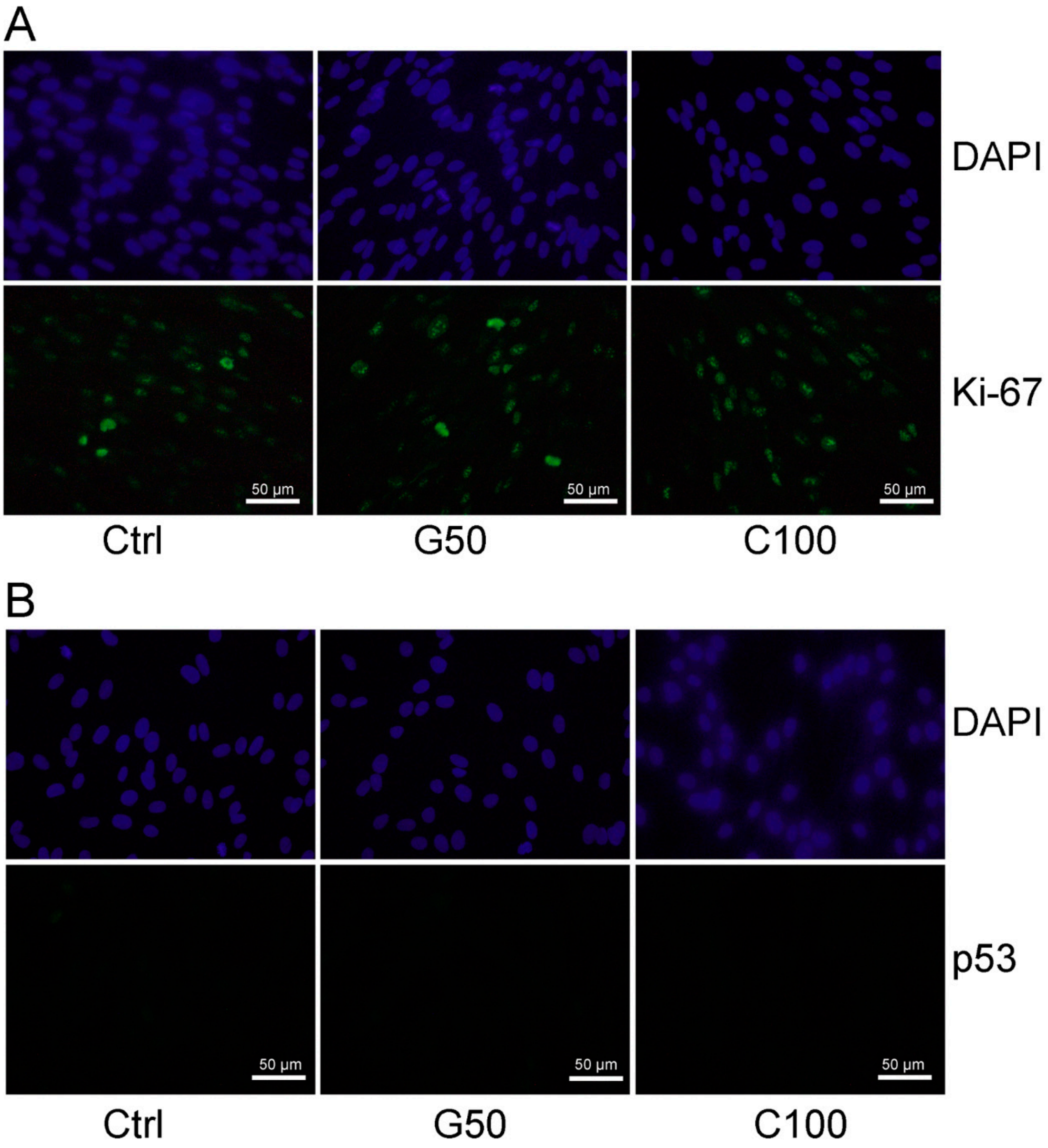

Supplementary Figure S2. Immunostaining of the hGFBs with Ki-67 (**A**) or p53 (**B**). The PFA-fixed hGFBs were immunostained with anti-Ki67 antibody (Ki-67), or anti-p53 antibody (DO-7) after the cells were treated with 50 μM genipin (G50) or 100 nM cytochalasin D (C100) for 48 hours. The cells stained with Ki-67 or p53 show in green colour, while nuclei are stained in blue with DAPI. Ctrl: control (untreated).

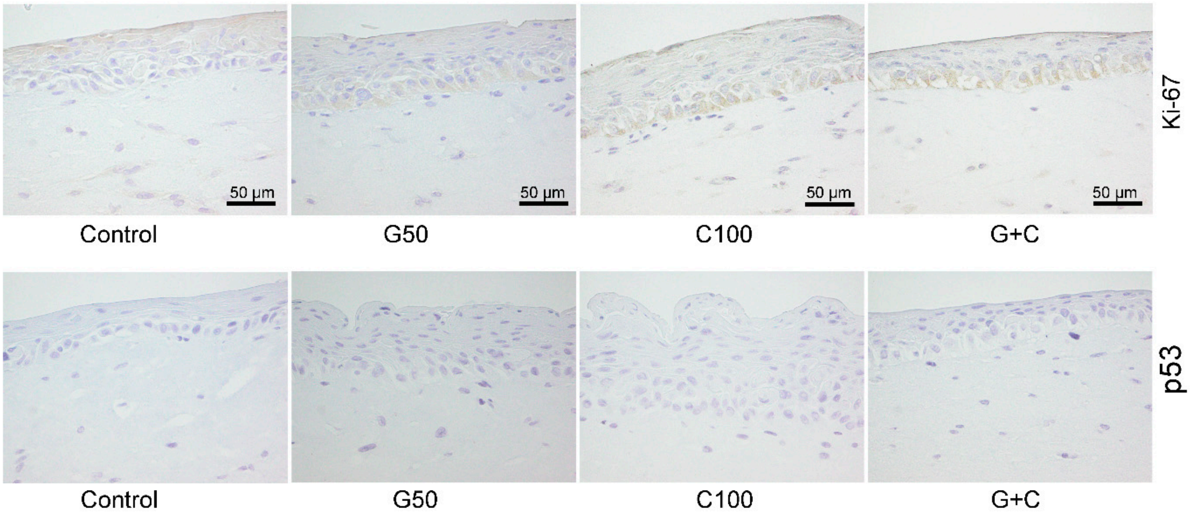

Supplementary Figure S3. Immunohistochemical (IHC) staining of Ki-67 and p53 in the human 3D gingival tissue equivalents (GTEs) generated with the TIGKs and the hGFBs-populated collagen gel with/without genipin/cytochalasin D directly crosslinking for 2-week air-liquid interface culture. Control: uncrosslinked 3D GTE; G50: 50 μM genipin; C100: 100 nM cytochalasin D; G+C: G50 + C100.

Supplementary Table S1. Significant p values of the hGFB size from multiple comparison between groups after the cells were treated with different concentrations of cytochalasin D in 2D monolayer culture.

Groups	Ctrl	C50	C100	C200	C400	C800
**Ctrl**		ns	ns	0.008	0.034	< 0.001
**C50**	ns		ns	0.001	0.004	< 0.001
**C100**	ns	ns		0.004	0.020	< 0.001
**C200**	0.008	0.001	0.004		ns	ns
**C400**	0.034	0.004	0.020	ns		0.01
**C800**	< 0.001	< 0.001	< 0.001	ns	0.01	

Ctrl: control; C50: 50 nM cytochalasin D; C100: 100 nM cytochalasin D; C200: 200 nM cytochalasin D; C400: 400 nM cytochalasin D; C800: 800 nM cytochalasin D.
